# The Immune Inhibitory Receptor LAIR-1 Is Highly Expressed by Plasmacytoid Dendritic Cells and Acts Complementary with NKp44 to Control IFNα Production

**DOI:** 10.1371/journal.pone.0015080

**Published:** 2010-11-30

**Authors:** Irene Bonaccorsi, Claudia Cantoni, Paolo Carrega, Daniela Oliveri, Gabrielle Lui, Romana Conte, Michele Navarra, Riccardo Cavaliere, Elisabetta Traggiai, Marco Gattorno, Alberto Martini, Maria Cristina Mingari, Alessandro Moretta, Guido Ferlazzo

**Affiliations:** 1 Laboratory of Immunology and Biotherapy, Department of Human Pathology, University of Messina, Messina, Italy; 2 Department of Experimental Medicine (DIMES), University of Genoa, Genoa, Italy; 3 Istituto Giannina Gaslini, Genoa, Italy; 4 Centro di Eccellenza per la Ricerca Biomedica (CERB), University of Genoa, Genoa, Italy; 5 Istituto Nazionale per la Ricerca sul Cancro, Genoa, Italy; 6 Pharmaco-Biological Department, University of Messina, Messina, Italy; 7 Department of Pediatrics, University of Genoa, Genoa, Italy; Centre de Recherche Public de la Santé (CRP-Santé), Luxembourg

## Abstract

Plasmacytoid dendritic cells (pDCs) are a subset of dendritic cells endowed with the capacity of producing large amounts of IFNα. Here we show that the Leukocyte-Associated Ig-like Receptor-1 (LAIR-1) is abundantly expressed on pDCs (the highest expression among all leukocytes) and its cross-linking inhibits IFNα production in response to Toll-like receptor ligands. Remarkably, LAIR-1 expression in pDCs is down-regulated in the presence of interleukin (IL)-3, thus indicating coordinated functions with NKp44, another pDC inhibitory receptor, which is conversely induced by IL-3. Nevertheless, the expression of NKp44 in pDCs isolated from secondary lymphoid organs, which is thought to be influenced by IL-3, is not coupled to a decreased expression of LAIR-1. Interestingly, pDCs isolated from peripheral blood of systemic lupus erithematosus (SLE) patients express lower levels of LAIR-1 while displaying slight but consistent expression of NKp44, usually undetectable on pDCs derived from healthy donors. Using sera derived from SLE patients, we show that LAIR-1 and NKp44 display synergistic inhibitory effects on IFNα production by interleukin IL-3 cultured pDCs stimulated with DNA immunocomplexes. In conclusion, our results indicate that the inhibitory function of LAIR-1 may play a relevant role in the mechanisms controlling IFNα production by pDCs both in normal and pathological innate immune responses.

## Introduction

Plasmacytoid dendritic cells (pDCs) constitute a distinct subset of dendritic cells present in lymphoid and non lymphoid tissues [1], [2] and characterized by the ability of producing large amounts of interferon (IFN)α upon stimulation by toll-like receptor (TLR)-7 and TLR-9 agonists [3]. Given the significance of type I IFNs in activating a wide range of cells of the innate and adaptive immune system [4], IFNα production has to be under tight control in order to prevent aberrant immune response that could harm the host. Although pDCs display an array of surface receptors able to modulate their response [5]–[9], the molecular mechanisms for the negative regulation of their activity have not yet been completely elucidated and some of them in fact exhibit peculiar features. For instance, IRp60 (CD300a), an inhibitory receptor expressed by different leukocytes [10], has been shown to play an unpredicted role when cross-linked on pDCs. Indeed, IRp60 triggering reduces, as expected, TNFα production but increases IFNα secretion by pDCs [11]. Another surface receptor, NKp44, is expressed on a subset of pDCs in tonsils and is inducible on PB pDCs after in vitro culture with interleukin (IL)-3 [12]. NKp44 has been originally identified as an NK activating receptor [13], [14] signalling through the ITAM-bearing DAP12 adaptor molecule [15]. Cross-linking of NKp44 on NK cells is associated with triggering of NK cell-mediated cytotoxicity. Paradoxically, cross-linking of NKp44 on pDCs does not trigger their functions but rather significantly inhibits IFNα production in response to TLR9 agonists, namely cytosine-phosphate-guanosine (CpG) oligonucleotides [12].

The expression of Leukocyte-Associated Ig-like Receptor-1 (LAIR-1), another relevant immune inhibitory receptor [16], [17], has not been investigated in pDCs so far. LAIR-1 recognizes a common collagen motif and contains a single extracellular Ig-like domain and two cytoplasmic tyrosine-based inhibitory motifs (ITIMs) that bind to the SH2 domain of phosphatases, leading to dephosphorylation of different kinases [18]. The inhibitory potential of LAIR-1 was demonstrated on several leukocyte subsets: cross-linking of LAIR-1 on human NK cells delivers a potent inhibitory signal that is capable of inhibiting target cell lysis mediated by resting and activated NK cells [16]–[19]. Similarly, LAIR-1 can inhibit the cytotoxic activity of effector T cells upon CD3 cross-linking or antigen stimulation [20]; also, LAIR-1 cross-linking leads to down-regulation of Ig and cytokine production in primary B cells [21] and inhibits the differentiation of peripheral blood precursors towards myeloid dendritic cells in vitro [22].

In this study, we show that the expression of LAIR-1 on pDCs is constitutively higher than on all other blood mononuclear cells and its cross-linking significantly inhibits IFNα production by pDCs stimulated with CpG oligonucleotides or DNA immunocomplexes. Remarkably, pDCs isolated from systemic lupus erithematosus (SLE) patients express lower levels of LAIR-1. Our data also suggest that this receptor displays coordinated regulatory functions in concert with NKp44 in order to restrain IFNα production by pDCs.

## Results

### LAIR-1 expression in plasmacytoid dendritic cells

**Figure 1:**
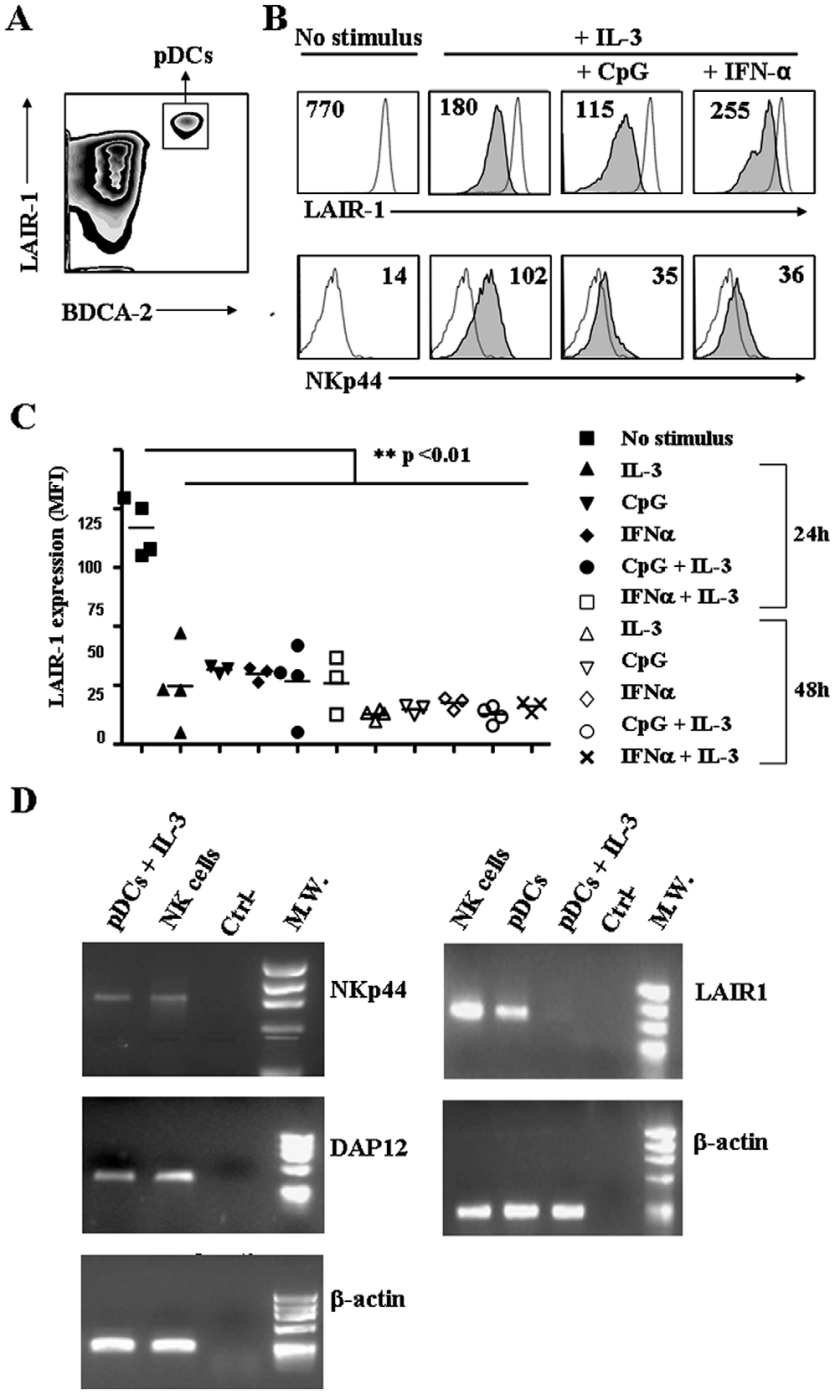
Expression of LAIR-1 and NKp44 on peripheral blood pDCs from healthy donors. Expression of LAIR-1 was assessed on pDCs from healthy donors PBMCs (A) and on purified pDCs (B) following culture in the presence of IL-3 (20 ng/ml), CpG (5 µg/ml) or IFNα (1000 U/ml) for 48 hours. (B): Dotted lines depict unstimulated cells; bold lines depict cells cultured as indicated. Values represent expression Geo-MFI. All experiments related to the analysis of LAIR-1 expression on pDCs cultured with the indicated stimuli for 24 and 48 hours are summarized in (C). Values depict Geo-MFI of positive cells/Geo-MFI of Ig isotype-matched control stained cells. (D): Analysis of LAIR-1, NKp44 and DAP12 mRNA expression on human pDCs. Polyclonal NK cell lines, pDCs and pDCs cultured 48 h with IL-3 were analyzed by RT-PCR for LAIR-1, NKp44 and DAP12 expression (see Materials and Methods).

Although LAIR-1 has been described in several immune cells, including subsets of myeloid dendritic cells, its expression has never been investigated in pDCs. Therefore, we first analyzed pDCs isolated from peripheral blood and found that LAIR-1 is constitutively highly expressed on circulating pDCs. Noteworthy, the expression of LAIR-1 in pDCs was consistently higher than on all other peripheral blood mononuclear cells (Figure 1A). Culturing pDCs in the presence of IL-3 resulted in down-regulation of LAIR-1 expression (Figure 1B,C,D). Similar down-regulation of LAIR-1 expression was observed on pDCs cultured either in the presence of TLR ligands or exogenous IFNα (Figure 1C) and the association of IL-3 with these latter stimuli did not further contribute to LAIR-1 down-regulation. As shown in figure 1D (right panel) after 48 h culture in the presence of IL-3, LAIR-1 mRNA was barely detectable.

Interestingly, LAIR-1 down-regulation in the presence of IL-3 is in sharp contrast with the expression of NKp44, another pDC inhibitory receptor that has been previously reported to be induced by IL-3 [12]. We thus confirmed IL-3-induced expression of NKp44 on pDCs and showed that also DAP12/KARAP mRNA, the NKp44-associated signalling adaptor protein, is regularly expressed by pDCs (Figure 1D, left panel). As previously reported [12], the association of IL-3 with CpG prevented the expression of NKp44 on pDCs. In addition, we now show that the expression of NKp44 induced by IL-3 can also be prevented by the presence of exogenous IFNα (Figure 1B, bottom panel).

### LAIR-1 engagement on pDCs induces inhibition of IFNα production

**Figure 2:**
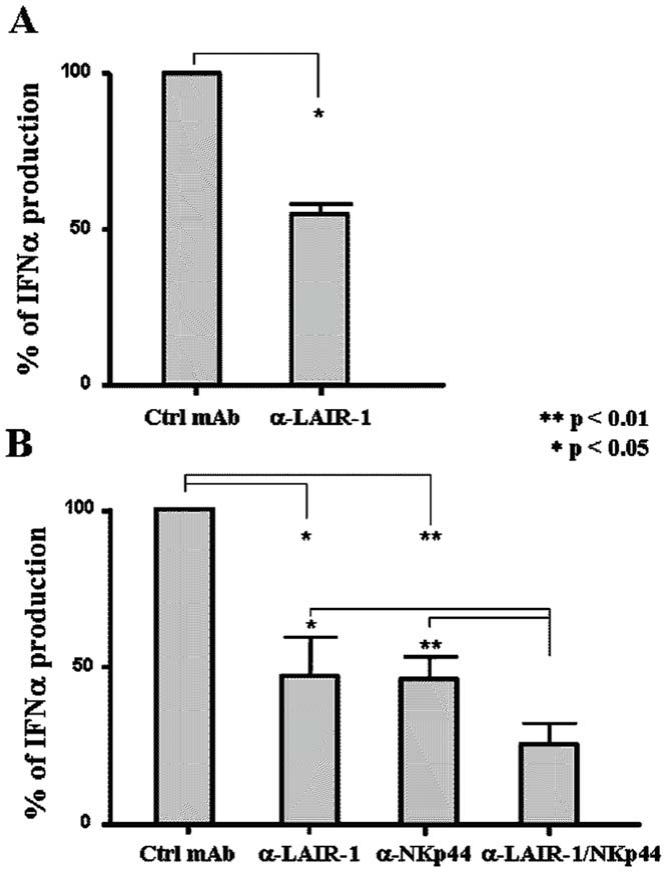
LAIR-1 triggering on pDCs induces inhibition of IFNα production. LAIR-1 cross-linking inhibits IFNα production by pDCs activated with either CpG (A) or DNA immunocomplexes (B). pDCs were cultured for 18 h in the presence of IL-3, incubated with the indicated mAbs or isotype-matched mAbs (Ctrl mAb) and then cultured in F(ab)2 goat anti-mouse IgG antibody-coated plates in the presence of CpG or DNA immunocomplexes. IFNα production was assessed by ELISA in culture supernatants. Graphs represent mean values +/− SEM of three independent experiments. ** p<0.01; * p<0.05.

We next investigated the functional effects of LAIR-1 triggering in pDCs. First, we analyzed the effect of LAIR-1 cross-linking on the production of IFNα by CpG ODN-activated pDCs. Our data indicate that the engagement of LAIR-1 significantly inhibited IFNα secretion in response to CpG stimulation (Figure 2A). Moreover, since the production of IFNα has been described to play a pivotal role in systemic lupus erithemathosus (SLE) as well as in the pathogenesis of other autoimmune disease, we investigated the effect of LAIR-1 cross-linking on pDCs stimulated with DNA/anti-DNA immunocomplexes. In these experiments, pDCs were stimulated with DNA from apoptotic cells in the presence of serum from SLE patients. This culture system has been previously shown to induce IFNα release by pDCs [23]. As shown in figure 2B, LAIR-1 cross-linking effectively inhibited IFNα production by pDCs in response to SLE serum-derived anti-DNA immune complexes. NKp44 was similarly able to inhibit IFNα production by pDCs stimulated by SLE anti-DNA immunocomplexes and, remarkably, consistently synergized with LAIR-1 for inhibiting IFNα release.

### LAIR-1 and NKp44 expression in peripheral tissue pDCs

**Figure 3:**
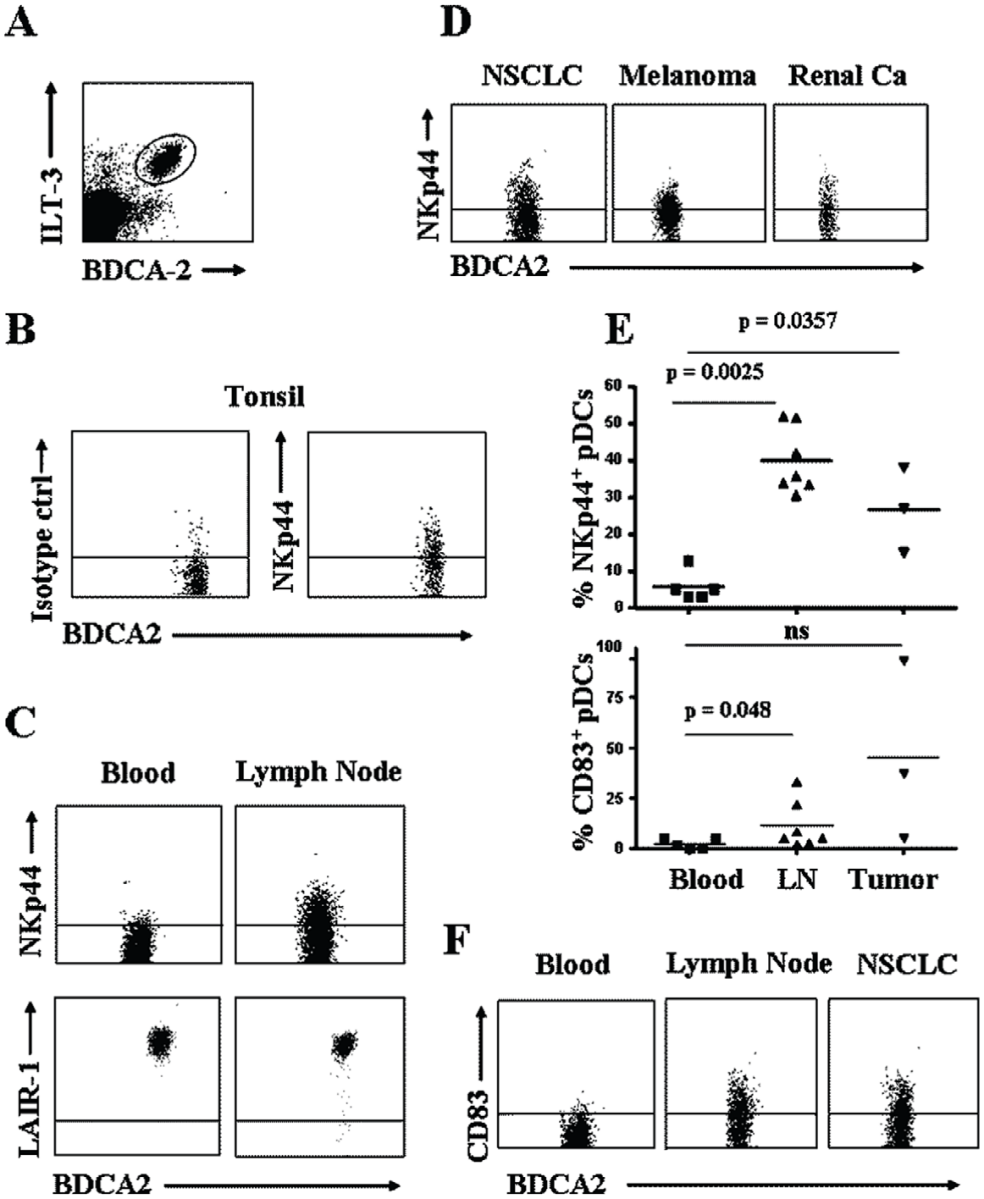
Expression of LAIR-1 and NKp44 on peripheral tissue pDCs. pDCs were identified in different tissues as BDCA-2^+^ ILT3^+^ positive cells (A). LAIR-1 and NKp44 expression was evaluated in tonsils (B), lymph nodes (C and E) and in different malignant tumors (D and E). pDCs activation in lymph nodes and in NSCLC was assessed as CD83 expression (E and F). The horizontal bars within dot-plots of panels C, D, F represent negative controls with isotype-matched irrelevant mAbs. Data shown are representative of at least three independent experiments analyzing pDCs from different patient tissues.

NKp44 has been reported to be expressed in a subset of tonsil pDCs and its expression was associated with the presence of IL-3 released by CD8^+^ memory T cells found in close proximity to pDCs [12]. In addition to inflamed tonsils (figure 3B), we have now extended the analysis also to non-reactive lymph nodes and found a similar expression of NKp44 in lymph node pDCs as well as in tumor-associated pDCs (Figure 3C,D,E). Tumor-associated pDCs were analyzed in non-small-cell-lung cancer (NSCLC), in renal cell carcinomas and melanomas (Figure 3D,E) and expression of NKp44 was detected in all tumors analyzed. These latter results will deserve further investigation since the ligand of NKp44, although still elusive, is known to be expressed on several cancer cell lines and might therefore impact on pDC function during the in vivo anti-tumor immune response. Moreover, different from peripheral blood pDCs, both lymph node and tumor-associated pDCs showed a discrete level of activation, assessed as surface CD83 expression (Figure 3E,F).

Since NKp44 expression on secondary lymphoid organs pDCs is assumed to be related to locoregional IL-3 production, we expected to detect, on the basis of our in vitro observations, a decreased expression of LAIR-1 in lymph node pDCs. However, as shown in figure 3C, the expression of LAIR-1 was similar in peripheral blood and autologous lymph node pDCs. Of note, the expression of BDCA-2, which is also reported to be down-regulated in the presence of IL-3 [5], [24], was comparable to that observed on autologous peripheral blood pDCs.

### Plasmacytoid DCs and Natural Killer cells share the same NKp44 protein sequence

In line with the reported inhibitory role of NKp44 in pDCs, we have shown that this receptor displays inhibitory activity also when pDCs are stimulated by anti-DNA immunocomplexes (Figure 2B), a model that more closely resembles the triggering of pDCs during autoimmune diseases. NKp44 has been initially identified as an activating receptor in NK cells and the molecular basis for this paradoxic behaviour in these two distinct leukocyte subsets has not yet been dissected. We therefore assessed whether the opposite functional outcomes of NKp44 triggering in NK cells and pDCs could be related to differences in the intracytoplasmic tail of the receptor. Thus, we amplified NKp44 cDNA from IL-3-treated healthy donor pDCs as well as from lymph node pDCs; analysis of nucleotide sequences demonstrated a complete identity with NKp44 molecule expressed on NK cells.

In addition, we analyzed nucleotide sequences of DAP12, the NKp44-associated adaptor protein, in pDCs and confirmed also in this case a complete identity with DAP12 expressed in NK cells.

### pDCs isolated from systemic lupus erithematosus patients express lower levels of LAIR-1

**Figure 4:**
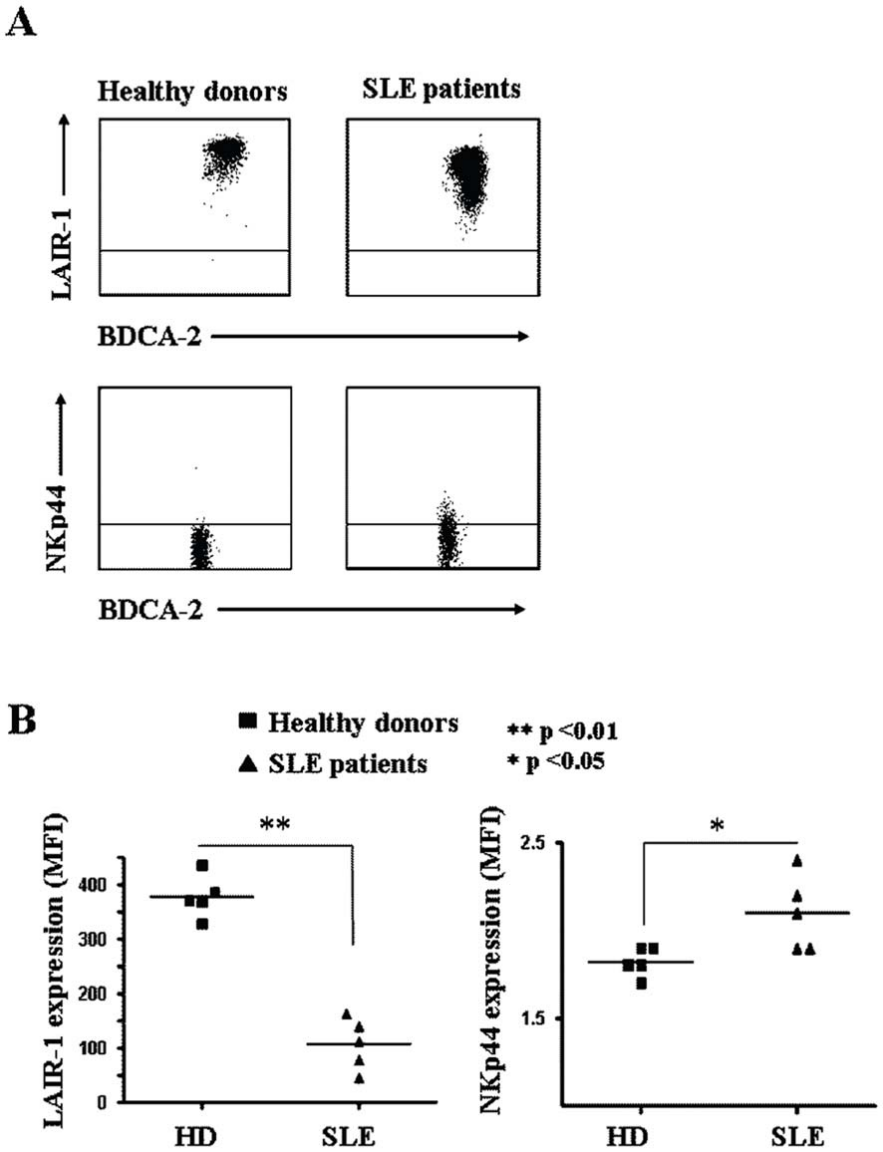
Expression of LAIR-1 and NKp44 on pDCs from SLE patients. PBMC samples were isolated from SLE patients and age matched healthy donors and the levels LAIR-1 and NKp44 expression on pDCs were investigated. A representative experiment is shown in panel A. The horizontal bars within the dot-plot depict negative controls with isotype-matched irrelevant mAbs. Results from different independent experiments are summarized in B. Geo-MFI index was used to compare the levels of LAIR-1 and NKp44 expression on pDCs and calculated as: Geo-MFI of positive cells/Geo-MFI of isotype staining cells.

It has been shown that systemic lupus erithematosus (SLE) patients exhibit elevated IFNα serum levels, which correlate with both disease activity and severity [25], [26]. Accordingly, activated pDCs, as a major source of IFNα, are possibly involved in the pathogenesis of SLE. Since we observed that in vitro pDC activation results in a decreased expression of LAIR-1, we investigated whether the expression of LAIR-1 was decreased on pDCs derived from SLE patients. To this aim, we collected peripheral blood of pediatric SLE patients and age-matched healthy donors and investigated the levels of LAIR-1 expression on pDCs. We found that the levels of LAIR-1 expression on pDCs isolated from SLE patients were significantly lower than those detectable on pDCs from healthy donors (Figure 4). On the other hand, we found that pDCs directly isolated from SLE patient PB display a slight but consistent expression of NKp44, which was rarely detectable in healthy donors.

## Discussion

pDCs are considered as the most potent “natural IFN-producing cells” [27] and, through production of this cytokine, they might activate multiple effector mechanisms of the immune system, including T cells, B cells and NK cells [3]. IFNα is a pivotal cytokine in antiviral immunity and is also associated with different autoimmune disorders such as lupus erythematosus, psoriasis, dermatomyositis and Sjogren's syndrome [1], [2], [28]. In SLE, pDCs might become chronically activated by antigen-antibody complexes containing RNA and DNA via TLR7 and 9, respectively [23]. Notably, IFNα levels are increased in sera of SLE patients and it has been postulated that this cytokine might be involved in myeloid DC activation during SLE disease [29]. As a consequence, IFNα producing pDCs have been suggested to be implicated in the pathogenesis of lupus disease [26]. Nevertheless, despite the relevance of IFNα in both normal and pathological immune response, the molecular mechanisms for the negative regulation of pDCs are not yet fully elucidated. Recently, novel pDC receptors able to modulate IFNα release have been described, such as NKp44 and IRp60 [11], [12]. NKp44 and IRp60 had been previously characterized as, respectively, activating and inhibitory receptors in other immune cell types [10], [13], [14]. Paradoxically, NKp44 acts on pDCs decreasing the release of IFNα in response to TLR-agonists, whereas IRp60 triggering results in an increase of IFNα production.

Here we show, for the first time, that LAIR-1 is expressed on pDCs and that its expression decreases along differentiation of pDCs in the presence of IL-3, as well as upon stimulation by CpG ODN, suggesting that pDCs exhibit decreased expression of LAIR-1 after activation. This confirms data reported in other immune cell types. Indeed, LAIR-1 expression was found to decrease in B lymphocytes upon cell activation in vitro [30]. Naïve B cells express high levels of LAIR-1 but the expression is absent in 50% of memory B cells and in plasma cells [30]. In T cells, LAIR-1 expression is high on naïve cells but lower and more heterogeneous on memory cells [31]. Thus, in several types of immune cells, a high cell surface expression of LAIR-1 is associated with a less differentiated phenotype, while LAIR-1 expression on memory and terminally differentiated cells is more heterogeneous.

We have demonstrated that mAb-mediated crosslinking of LAIR-1 sharply inhibits the release of IFNα by pDC stimulated by TLR ligands. Therefore, the strong inhibitory function previously assigned to LAIR-1 in studies focused on other leukocyte subsets, has been confirmed in the present analysis of pDC function.

Interestingly, exposure of pDCs to exogenous IL-3 results in two opposite effects: down-modulation of LAIR-1 and induction of NKp44 expression. Since triggering of both NKp44 and LAIR-1 on pDCs results in the inhibition of IFNα release upon TLR ligand stimulation, a cytokine-regulated coordinated function of LAIR-1 and NKp44 can be envisaged.

We also demonstrated that the presence of IFNα decreases the expression of LAIR-1 on pDCs and concomitantly inhibits IL-3-induced expression of NKp44. Similarly, also IRp60 expression on pDCs was recently described to be down-regulated by TLR ligands and this effect was associated to the release of IFNα by pDCs. Thus, CpG might also induce a decreased expression of both LAIR-1 and NKp44 via the release of IFNα. However, while IFNα can be envisioned as a mechanism of negative feedback, the functional significance of the down-regulation of two pDC inhibitory receptors by IFNα remains uncertain.

NKp44 expression on pDCs has been previously associated to the presence of IL-3 released by memory CD8^+^ T cells, found in close proximity to pDCs in tonsils [12]. Nevertheless, we now show that the expression of NKp44 in pDCs of secondary lymphoid organs, such as lymph nodes and tonsils, is not coupled to a decreased expression of LAIR-1. This may suggest that in secondary lymphoid organs, besides IL-3, other factors might induce the expression of NKp44 on pDCs without affecting the levels of LAIR-1 expression.

Given the potential role of IFNα in SLE pathogenesis, we assessed whether cross-linking of LAIR-1 or NKp44 might affect IFNα production by pDCs stimulated not only with CpG but also with DNA/SLE anti-DNA immune complexes. Our results demonstrate that cross-linking of LAIR-1 effectively inhibited IFNα production by pDCs in response to DNA immunocomplexes. Remarkably, also NKp44 effectively inhibited IFNα production by pDCs in response to these stimuli and consistently synergized with LAIR-1 to provide optimal inhibitory function.

Having confirmed the inhibitory activity of NKp44 also in pDCs stimulated with DNA immunocomplexes, we wondered whether NKp44 molecule expressed by pDCs might differ from that expressed by NK cells (where the same receptor activates NK cell functions), hypothesizing, for instance, differences in its intracytoplasmic tail. Indeed, nucleotide sequence analysis demonstrated a complete identity of both NKp44 and the related adaptor protein DAP12 with the same molecules expressed on NK cells. Thus, the signalling mechanisms ruling this contrasting behaviour in the two cell types remains yet unknown. One possibility relates to alternative signalling delivered via DAP12, which contains immunoreceptor tyrosine-based activating motif (ITAM), which recruits protein tyrosine kinases [32]. As a matter of fact, in some instances, it has been reported that ITAMs can recruit tyrosine phosphatases instead of tyrosine kinases and therefore mediate inhibition [12], [15], [33]. On the other hand, another explanation may reside in NKp44-mediated release of yet unidentified cytokines able to inhibit IFNα production.

We also found that pDCs from SLE patients express lower amounts of LAIR-1 as compared to age-matched healthy donors. The impaired expression in SLE patients most likely reflects the activation of pDCs, which has been suggested to occur in these patients because of anti-nucleic acids immune complexes [23]. However, in the same SLE patients cohort, also lymphocytes displayed lower levels of LAIR-1 (not shown), which might be similarly related to their in vivo activation [31]. Nevertheless, another intriguing scenario may envisage an inherited deficiency of one or more inhibitory receptors that might be involved in the pathogenesis of the autoimmune disease. However, this latter hypothesis contrasts with the slight but consistent expression of NKp44 observed on SLE PB pDCs, which also points to an activation status of pDCs in SLE patients. Whatever the case, a persistently impaired expression of pDC inhibitory receptors, such as LAIR-1 or BDCA-2 [24], might contribute to the maintenance of elevated IFNα levels and therefore to the pathologic immune response.

In conclusion, we have described the expression of the immune inhibitory receptor LAIR-1 on pDCs and showed that, in a coordinated fashion with NKp44, it is able to control the release of IFNα in response to TLR ligands, including anti-DNA immuno complexes. In addition, we extended previous knowledge about NKp44 function in pDCs of peripheral blood and tissues. A deeper understanding of the mechanisms ruling type I IFN production and, more specifically, of pDC functional receptors, will be fundamental for planning new immune interventions for controlling autoimmune, viral and neoplastic diseases.

## Materials and Methods

### Ethics Statement

This study was approved by the Ethics Committee of Istituto Nazionale per la Ricerca sul Cancro, Genoa (in order to obtain cancer patient blood and tissues) and of Istituto G.Gaslini, Genoa (in order to obtain peripheral blood sera and cells of SLE patients). All patients gave written informed consent according to the Declaration of Helsinki.

### Monoclonal antibodies and flow cytometry

The following mAbs were employed for pDC identification and analysis: allophycocyanin- or FITC-conjugated anti-BDCA-2 (clone AC144) (Miltenyi Biotec, Bergish Gladbach, Germany), anti-LAIR-1 (clone 1F1) and anti-NKp44 (clone Z231 and KS38) produced in our laboratory, PC5-conjugated anti-ILT3 (clone ZM3.8) and PE-conjugated anti-CD83 (clone HB15A) (Beckman Coulter, Immunotech, Marseille, Cedex, France).

For analysis of pDCs in secondary lymphoid organ and tumors, cells were labeled with anti-ILT-3 and BDCA-2 mAbs and analysis was performed on ILT3^+^ and BDCA-2^+^ cells. For indirect immunofluorescence staining, the relevant mAb was added and incubated for 30 min at 4°C. After extensive washing, FITC- or PE-conjugated isotype-specific goat anti-mouse mAbs (Southern Biotechnology Associates, Birmingham, AL) were added and incubated for 30 min at 4°C. Negative controls included directly labeled or unlabeled isotype-matched irrelevant mAbs.

Analysis were carried out by FACSCanto II flow cytometer (Becton Dickinson, Mountain View, CA). Data analysis was performed using Flowjo software 7.5.3 (Tree Star).

### pDC isolation, culture and tissue processing

Human PBMCs were prepared by Ficoll-Hypaque (Pharmacia, Uppsala, Sweden) density gradient centrifugation of buffy coats from normal donors (Blood Center, A.O.U., Messina) or SLE patient whole blood and corresponding healthy controls (Istituto G. Gaslini, Genoa). pDCs were obtained from PBMCs by using MACS magnetic labelling system (Miltenyi Biotec, Bergish Gladbach, Germany). Briefly, pDCs were purified by negative selection using pDC isolation kit and MACS LD columns (Miltenyi Biotec). Purity was at least 97%. Purified pDCs were cultured in RPMI 1640 (Euroclone, Milan, Italy), supplemented with 10% fetal bovine serum (Euroclone, Milan, Italy), penicillin (60 mg/ml), streptomycin (100 mg/ml), L-glutamine (2 mM) and IL-3 (20 ng/mL) (all from R&D Systems, Minneapolis, MN). For pDCs stimulation, 5 µg/mL CpG ODN-A (TIB Molbiol, Genoa, Italy) or IFNα (1000 U/ml) (Schering Plough) were added to cultures at the indicated time point (24 or 48 hours). Secondary lymphoid organs and tumor tissue were processed as previously described [34], [35]. Briefly, secondary lymphoid organs were mechanically dissociated and then filtered through a 100 µ nylon cell strainer (BD Labware, Mountain View, CA) to exclude undissociated fragments. Debris and dead cells were eliminated using a Ficoll-Hypaque discontinuous gradient. Tumor tissues were processed by mechanical dissociation followed by enzymatic digestion. the down-regulation of IRp60 on pDCs by.

### IFNα detection following stimulation and receptor cross-linking

For receptor cross-linking experiments, pDCs were labelled with saturating amount of the indicated mAbs and then cultured in 96-well plates coated with F(ab)2 goat anti mouse IgG antibody (10 µg/mL; Southern Biotechnology Associates, Birmingham, AL). 5×10^4^ pDCs/well were cultured with 2,5% human sera, penicillin (60 mg/ml), streptomycin (100 mg/ml), L-glutamine (2 mM), IL-3 (20 ng/mg) and CpG ODN-A (5 µg/ml; Tib MolBiol, Genoa) in a final volume of 0,1 ml. After 48 h culture supernatants were harvested for cytokine analysis. Alternatively, for stimulation by DNA immunocomplexes, 5×10^4^ pDCs were cultured in RPMI supplemented with 5×10^4^ γ-irradiated U937 cells, in the presence of 13% SLE serum, as previously described [23].

For NKp44 cross-linking experiments pDCs were previously cultured for 18 h in the presence of IL-3 (20 ng/mL; R&D Systems, Minneapolis, MN) in order to induce NKp44 expression.

The levels of IFNα in culture supernatants were determined by an ELISA kit according to manufactures'instructions (R&D System).

### RT-PCR analysis and sequencing

Total RNA was extracted from polyclonal NK cell populations, pDC and pDC cultured for 48 h in the presence of IL-3 using RNAeasy Micro Kit (Qiagen, Hilden, Germany). Oligo (dT)-primed cDNA was prepared by standard technique using Transcriptor (Roche, Monza, Italy). PCR amplifications were performed with the following primers: β-actin up ACTCCATCATGAAGTGTGACG; β-actin dw CATACTCCTGCTTGCTGATCC; LAIR-1 ORF up 5′ GTATGGGGTCAGTGTCTGG; LAIR-1 ORF dw 5′ CATTGGTGCGCCTCAGGC; NKp44 ORF up 5′ CCACGAGCGCACAGGAAAAGG; NKp44 ORF dw 5′ TCACAAAGTGTGTTCATCATCATCATCGCTTATCTTAGTCC; DAP12 up 5′ TCATGGGGGGACTTGAACC; DAP12 dw 5′ GATTCGGGCTCATTTGTAATAC. Amplifications were performed utilizing Platinum TAQ (Invitrogen, Carlsbad, CA) for 30 cycles (30 sec. at 95°C, 30 sec. at 58°C, 1 min. at 68°C) except for NKp44 for which the annealing temperature was 65°C. PCR products (249 bp fragment for β-actin, 916 bp for LAIR-1, 857 bp for NKp44 and 353 bp for DAP12) were run on a 0.8% agarose gel and visualized by ethidium bromide staining. LAIR-1, NKp44 and DAP12 PCR products amplified from pDC were subcloned in pcDNA3.1/V5-His TOPO vector (Invitrogen, Carlsbad, CA). DNA sequencing was performed using BigDye Terminator Cycle Sequencing Kit and a 377 Applied Biosystems Automatic Sequencer (Perkin Elmer-Applied Biosystems).

### Statistical analysis

The significance of difference between groups was calculated by t-test using GraphPad Prism 4.0 software (GraphPad Software, San Diego, CA). *P* values less than or equal to 0,05 were considered significant.

## References

[1] Gilliet M, Cao W, Liu YJ (2008). Plasmacytoid dendritic cells: sensing nucleic acids in viral infection and autoimmune diseases.. Nat Rev Immunol.

[2] Sozzani S, Vermi W, Del Prete A, Facchetti F (2010). Trafficking properties of plasmacytoid dendritic cells in health and disease.. Trends Immunol.

[3] Kadowaki N, Antonenko S, Liu YJ (2001). Distinct CpG DNA and polyinosinic-polycytidylic acid double-stranded RNA, respectively, stimulate CD11c^−^ type 2 dendritic cell precursors and CD11c^+^ dendritic cells to produce type I IFN.. J Immunol.

[4] Banchereau J, Pascual V (2006). Type I interferon in systemic lupus erythematosus and other autoimmune diseases.. Immunity.

[5] Dzionek A, Sohma Y, Nagafune J, Cella M, Colonna M (2001). BDCA-2, a novel plasmacytoid dendritic cell-specific type II C-type lectin, mediates antigen capture and is a potent inhibitor of interferon alpha/beta induction.. J Exp Med.

[6] Bave U, Magnusson M, Eloranta ML, Perers A, Alm GV (2003). Fc gamma RIIa is expressed on natural IFN-alpha-producing cells (plasmacytoid dendritic cells) and is required for the IFN-alpha production induced by apoptotic cells combined with lupus IgG.. J Immunol.

[7] Blasius AL, Cella M, Maldonado J, Takai T, Colonna M (2006). Siglec-H is an IPC-specific receptor that modulates type I IFN secretion through DAP12.. Blood.

[8] Cao W, Rosen DB, Ito T, Bover L, Bao M (2006). Plasmacytoid dendritic cell-specific receptor ILT7-Fc epsilonRI gamma inhibits Toll-like receptor-induced interferon production.. J Exp Med.

[9] Fanning SL, George TC, Feng D, Feldman SB, Megjugorac NJ (2006). Receptor cross-linking on human plasmacytoid dendritic cells leads to the regulation of IFN-alpha production.. J Immunol.

[10] Cantoni C, Bottino C, Augugliaro R, Morelli L, Marcenaro E (1999). Molecular and functional characterization of IRp60, a member of the immunoglobulin superfamily that functions as an inhibitory receptor in human NK cells.. Eur J Immunol.

[11] Ju X, Zenke M, Hart DN, Clark GJ (2008). CD300a/c regulate type I interferon and TNF-alpha secretion by human plasmacytoid dendritic cells stimulated with TLR7 and TLR9 ligands.. Blood.

[12] Fuchs A, Cella M, Kondo T, Colonna M (2005). Paradoxic inhibition of human natural interferon-producing cells by the activating receptor NKp44.. Blood.

[13] Vitale M, Bottino C, Sivori S, Sanseverino L, Castriconi R (1998). NKp44, a novel triggering surface molecule specifically expressed by activated natural killer cells, isinvolved in non-major histocompatibility complex-restricted tumor cell lysis.. J Exp Med.

[14] Cantoni C, Bottino C, Vitale M, Pessino A, Augugliaro R (1999). NKp44, a triggering receptor involved in tumor cell lysis by activated human natural killer cells, is a novel member of the immunoglobulin superfamily.. J Exp Med.

[15] Campbell KS, Yusa S, Kikuchi-Maki A, Catina TL (2004). NKp44 triggers NK cell activation through DAP12 association that is not influenced by a putative cytoplasmic inhibitory sequence.. J Immunol.

[16] Poggi A, Pella N, Morelli L, Spada F, Revello V (1995). p40, a novel surface molecule involved in the regulation of the non-major histocompatibility complex-restricted cytolytic activity in humans.. Eur J Immunol.

[17] Meyaard L, Adema GJ, Chang C, Woollatt E, Sutherland GR (1997). LAIR-1, a novel inhibitory receptor expressed on human mononuclear leukocytes.. Immunity.

[18] Meyaard L (2008). The inhibitory collagen receptor LAIR-1 (CD305).. J Leukoc Biol.

[19] Poggi A, Tomasello E, Revello V, Nanni L, Costa P (1997). p40 molecule regulates NK cell activation mediated by NK receptors for HLA class I antigens and TCR-mediated triggering of T lymphocytes.. Int Immunol.

[20] Meyaard L, Hurenkamp J, Clevers H, Lanier LL, Phillips JH (1999). Leukocyte-associated Ig-like receptor-1 functions as an inhibitory receptor on cytotoxic T cells.. J Immunol.

[21] Merlo A, Tenca C, Fais F, Battini L, Ciccone E (2005). Inhibitory receptors CD85j, LAIR-1, and CD152 down-regulate immunoglobulin and cytokine production by human B lymphocytes.. Clin Diagn Lab Immunol.

[22] Poggi A, Tomasello E, Ferrero E, Zocchi MR, Moretta L (1998). p40/LAIR-1 regulates the differentiation of peripheral blood precursors to dendritic cells induced by granulocyte-monocyte colony-stimulating factor.. Eur J Immunol.

[23] Bave U, Alm GV, Ronnblom L (2000). The combination of apoptotic U937 cells and lupus IgG is a potent IFN-alpha inducer.. J Immunol.

[24] Wu P, Wu J, Liu S, Han X, Lu J (2008). TLR9/TLR7-triggered downregulation of BDCA2 expression on human plasmacytoid dendritic cells from healthy individuals and lupus patients.. Clin Immunol.

[25] Pascual V, Farkas L, Banchereau J (2006). Systemic lupus erythematosus: all roads lead to type I interferons.. Curr Opin Immunol.

[26] Ronnblom L, Pascual V (2008). The innate immune system in SLE: type I interferons and dendritic cells.. Lupus.

[27] Colonna M, Trinchieri G, Liu YJ (2004). Plasmacytoid dendritic cells in immunity.. Nat Immunol.

[28] Albanesi C, Scarponi C, Pallotta S, Daniele R, Bosisio D (2009). Chemerin expression marks early psoriatic skin lesions and correlates with plasmacytoid dendritic cell recruitment.. J Exp Med.

[29] Blanco P, Palucka AK, Gill M, Pascual V, Banchereau J (2001). Induction of dendritic cell differentiation by IFN-alpha in systemic lupus erythematosus.. Science.

[30] van der Vuurst de Vries AR, Clevers H, Logtenberg T, Meyaard L (1999). Leukocyte-associated immunoglobulin-like receptor-1 (LAIR-1) is differentially expressed during human B cell differentiation and inhibits B cell receptor-mediated signaling.. Eur J Immunol.

[31] Jansen CA, Cruijsen CW, de Ruiter T, Nanlohy N, Willems N (2007). Regulated expression of the inhibitory receptor LAIR-1 on human peripheral T cells during T cell activation and differentiation.. Eur J Immunol.

[32] Vivier E, Nunes JA, Vely F (2004). Natural killer cell signaling pathways.. Science.

[33] Pasquier B, Launay P, Kanamaru Y, Moura IC, Pfirsch S (2005). Identification of FcalphaRI as an inhibitory receptor that controls inflammation: dual role of FcRgamma ITAM.. Immunity.

[34] Ferlazzo G, Pack M, Thomas D, Paludan C, Schmid D (2004). Distinct roles of IL-12 and IL-15 in human natural killer cell activation by dendritic cells from secondary lymphoid organs.. Proc Natl Acad Sci U S A.

[35] Carrega P, Morandi B, Costa R, Frumento G, Forte G (2008). Natural killer cells infiltrating human nonsmall-cell lung cancer are enriched in CD56 bright CD16(-) cells and display an impaired capability to kill tumor cells.. Cancer.

